# The *Anopheles dirus *complex: spatial distribution and environmental drivers

**DOI:** 10.1186/1475-2875-6-26

**Published:** 2007-03-06

**Authors:** Valérie Obsomer, Pierre Defourny, Marc Coosemans

**Affiliations:** 1 Department of Parasitology, Prince Leopold Institute of Tropical Medicine, Nationalestraat 155, 2000 Antwerp, Belgium; 2 Department of Environmental Sciences and Land Use Planning, Université Catholique de Louvain, Croix du Sud 2/16, B-1348 Louvain-la-Neuve, Belgium

## Abstract

**Background:**

The *Anopheles dirus *complex includes efficient malaria vectors of the Asian forested zone. Studies suggest ecological and biological differences between the species of the complex but variations within species suggest possible environmental influences. Behavioural variation might determine vector capacity and adaptation to changing environment. It is thus necessary to clarify the species distributions and the influences of environment on behavioural heterogeneity.

**Methods:**

A literature review highlights variation between species, influences of environmental drivers, and consequences on vector status and control. The localisation of collection sites from the literature and from a recent project (MALVECASIA) produces detailed species distributions maps. These facilitate species identification and analysis of environmental influences.

**Results:**

The maps give a good overview of species distributions. If species status partly explains behavioural heterogeneity, occurrence and vectorial status, some environmental drivers have at least the same importance. Those include rainfall, temperature, humidity, shade, soil type, water chemistry and moon phase. Most factors are probably constantly favourable in forest. Biological specificities, behaviour and high human-vector contact in the forest can explain the association of this complex with high malaria prevalence, multi-drug resistant *Plasmodium falciparum *and partial control failure of forest malaria in Southeast Asia.

**Conclusion:**

Environmental and human factors seem better than species specificities at explaining behavioural heterogeneity. Although forest seems essential for mosquito survival, adaptations to orchards and wells have been recorded. Understanding the relationship between landscape components and mosquito population is a priority in foreseeing the influence of land-cover changes on malaria occurrence and in shaping control strategies for the future.

## Background

Throughout most of their geographical distribution, species of the *Anopheles dirus *complex are associated with high malaria prevalence rates and the occurrence of drug resistant *Plasmodium falciparum *[1,2]. The biological specificities of these efficient vectors undermine the most popular control measures and challenge the success of malaria control. Sporadic studies on sympatric sibling species from the complex suggest ecological and biological differences in types of larval habitat, seasonality and behaviour according to species [3-5] but such differences also occur for specific species within their distributions and could relate to key environmental factors. Variation in behaviour, such as early biting or ovipositing in wells, might determine vector capacity and adaptation to changing environment. It is thus of interest to clarify the geographical distributions of the species, the importance of environmental factors and the influences of ecological variation on behavioural heterogeneity.

If the biology of *An.dirus s.l*. (*sensu lato*, i.e. *An. dirus *complex) is well documented in the literature, no attempt has been made recently to compile this information. A major difficulty resides in the taxonomic changes which have affected the group throughout the last 50 years [4,6,7]. Furthermore, only recently did molecular tools allow identification of individuals from this group up to the species level and they rely on strenuous methods or require sophisticated equipment. As a result, most of the available articles do not provide specific species identification.

**Figure 1:**
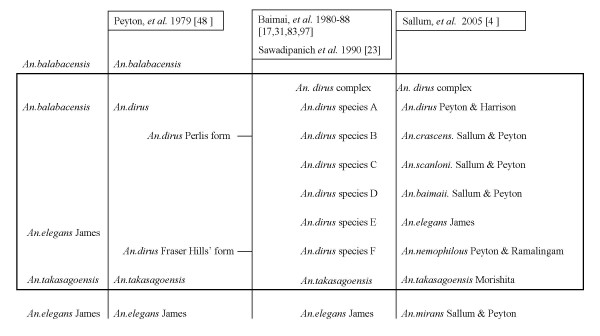
**The *Anopheles dirus *complex**. Taxonomic history of the *Anopheles dirus *complex and correspondence with historical names, including milestone articles.

Fortunately, the taxonomy of the complex has recently been clarified and the species named [4,8]. The complex belongs to the *Anopheles (Cellia) leucosphyrus *group in the Neomyzomyia Series [6] and now includes at least seven species: *Anopheles dirus *or *An. dirus sensu stricto *(*s.s*.), *Anopheles crascens*, *Anopheles scanloni*, *Anopheles baimaii*, *Anopheles elegans (*previously known as species E), *Anopheles nemophilous *and *Anopheles takasagoensis *[4,8]. The species previously called *Anopheles elegans *from Sri Lanka [9] and Southern India [10] has now been renamed *Anopheles mirans *and is not part of the complex [4]. Evolution of the complex and correspondence with historical names are presented in Figure 1.

Based on those taxonomic clarifications, existing distribution maps of these species [11] can now be updated using data from collection sites available in the literature and from the recent project MALVECASIA on monitoring insecticide resistance in Southeast Asia. Localisation of collection sites can facilitate identification of the species in articles lacking this information and provide the background for analysis of spatial distribution, biology, behaviour, vectorial status and key ecological factors of individual species.

This article thus aims to, 1) update the distributions of members of the *An. dirus *complex using literature records and personal data, 2) to provide an overview of intra- and inter- species variation of biology and behaviour and 3) to identify key ecological factors influencing the behaviour distribution, occurrence and vectorial status of *An. dirus s.l*.

## Methods

The basis for the paper is a comprehensive analysis of more than a hundred articles examined for the following items: information on geographical dispersion, species identification methods, behaviour of adults and larvae, and environmental factors influencing occurrence and behaviour. Key papers on taxonomy and population structure were first reviewed to associate historical species records with currently recognized species.

More than five hundred collection sites were then spatially located. They include literature records but also recent data from the MALVECASIA network, a research network of eight partners that studied the distribution and insecticide resistance of malaria vectors in Vietnam, Cambodia, Laos and Thailand. This information was gathered in a table, and maps were produced to plot the collection sites on a vegetation background adapted from the global dataset Global Land Cover 2000[12]. This background represents what is thought to be a forested habitat from a mosquito's point of view. It includes the following original classes: evergreen and deciduous forest, open or closed, including mixed leaf type, flooded forest, mosaic of tree cover and other natural vegetation as well as mosaic of cropland, tree cover and other natural vegetation. The accuracy of site location depends on the available information: the maximum accuracy is obtained when the coordinates are provided in the original article. In most of the other cases, the extended Geographical Information System (SEAGIS) gathered and organized by the MALVECASIA network [13] provided the necessary tools to find an accurate location. Maps or detailed site descriptions were compared with datasets such as village databases, administrative maps, roads, rivers, vegetation and altitude. Some collection sites could not be accurately located using the above mentioned methods which made it necessary to look for locations in gazetteers [14].

For each site number, the sibling species identified in the reviewed publication is recorded, as well as the identification methods. Considerable uncertainty may result from some of these methods [15] and the distinction between *An. dirus s.s*. and *An. scanloni *remains problematic. Morphological keys based on reared adults with associated larval and pupal exuviae[16], polytene chromosomal banding patterns[17], enzyme electromorph [18], allele-specific polymerase chain reaction (ASPCR) [15,19] and random amplified polymorphic DNA (RAPD) [20] are a few of the existing identification methods. Sallum *et al*. provided a complete list [4]. Species status can be extrapolated from the map, for sites located in allopatric zones.

The species spatial distribution is first discussed. Then larval and adult ecology and behaviour are analysed to highlight possible variation between species. The influence of environmental drivers and land-cover is then considered as well as the consequences for vector status and control.

## Results and Discussion

### Species spatial distribution

**Figure 2:**
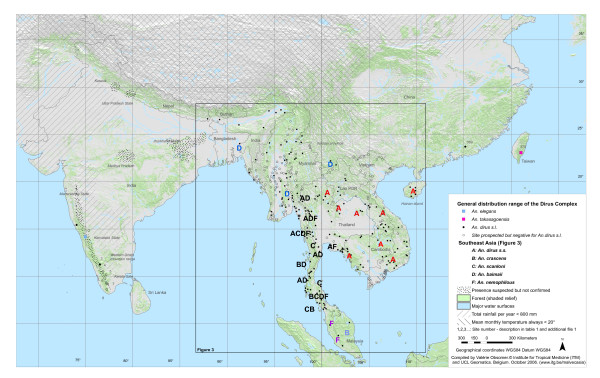
**Detailed distribution of the species from the *Anopheles dirus *complex**. Distribution of the sibling species of the *Anopheles dirus *complex depicted on a vegetation background and some indication of important temperature and rainfall thresholds. Each point represents one or several collection sites from the literature and from personal recent data. Details for each site are available in table 1 and in the additional file provided with this article. The details for the Southeast Asian region are shown in figure 3.

**Figure 3:**
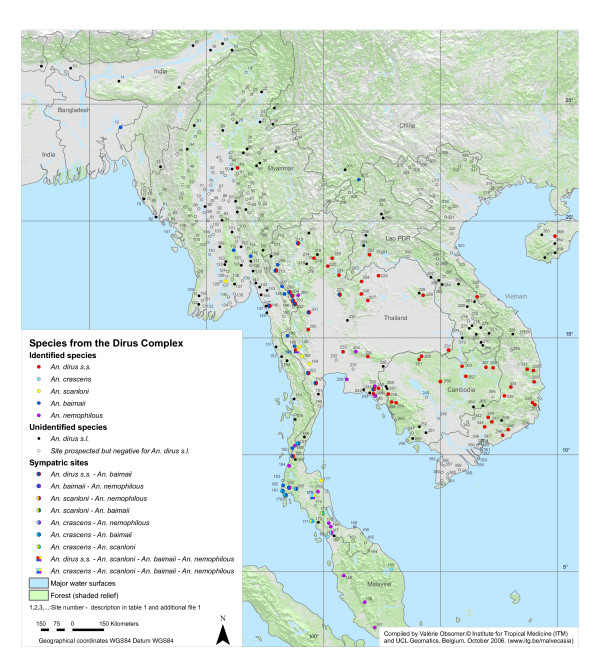
**Species from the *Anopheles dirus *complex in the Southeast Asian region**. Distribution of the sibling species of the *Anopheles dirus *complex depicted on a vegetation background. Each point represents one or several collection sites from the literature and from personal recent data. Details for each site are available in table 1 and in the additional file provided with this article.

A map first shows the overall extent of the *An. dirus *complex distribution (Figure 2). A second map focuses on Southeast Asia, where the species diversity and the number of records are higher (Figure 3). In this area, the presence of *An. dirus s.l*. seems to correspond to presence of malaria cases. The additional file 1 allows better reading of the maps. It indicates the reviewed publication, the species discovered and the identification method used for each site. As it was not graphically possible to display individually the more than 500 sites, sites close to each other are represented by a single point on the map and a single reference number. The additional files (Additional file 2, 3 and 4) associated with the current article provide a description of the site, collection methods and the number of *An. dirus s.l*. collected, as well as the original location for each of the 500 sites organized by site number or by reviewed publication. The current article is also aimed at malaria control workers, and with this information, they can find the situation recorded at any of the collection sites in the past, as well as locate any collection site cited in the publications reviewed.

Species of the *An. dirus *complex can be found in forest and forest foothills from India to Taiwan and from the 30° north parallel to the Malaysian peninsular. The mean monthly temperature below 20° seems to limit the northern distribution of the complex to just beyond the border of India with Nepal and Bhutan. Rainfall is probably the limiting factor to the west with annual rainfall per year under 800 mm. The absence of species of the complex in large non-forested areas of Thailand, southern Vietnam and central India is probably linked to the lack of suitable habitat. However the species of the complex are also absent from the north of Vietnam. This is most peculiar, given that this area is still forested and that members of the complex occur at the same latitude in neighbouring countries. The possible occurrence of *An. dirus s.l*. in central India was investigated by Srivastava [21]. A predictive model identifying suitable factors for *Anopheles *survival and the distribution of wet evergreen and deciduous forest confirmed the presence of *An. dirus s.l*. in already well-known areas of occurrence, but also in a few suitable sites through central and North-Western India. This seems to correspond to historical collection sites in Uttar Pradesh, Kerala and Karnakata. Bhat [22] and Rajavel [7] also cite historical records in Kasauli and other regions now considered free of *An. dirus s.l*. Recent reports of the presence of *An. dirus s.l*. in the state of Jharkhand (Dr. Diwakar Dinesh, personal communication) call for further investigation into the actual distribution of the complex in India. Those areas are presented in Figure 2 as sites with presence suspected but not confirmed.

Regarding the species distributions within the range of the complex, there are no major geographic or topographic reasons that seem to justify the current species distribution pattern. Therefore, speciation probably does not result from adaptation to a specific environment but the species might have been isolated for some time. Their distribution most probably reflects geo-morphological changes which have occurred in the past and are today not evident [3]. The mountains of the Western Ghats in southern India are the most westward limit of the complex with the presence of *An. elegans *[10,23] apparently isolated from the other species. The closest records to this area come then from the north eastern states in India [24] and the border of Nepal [25], and are considered to be *An. baimaii *based on identification in nearby Bangladesh [26]. *An. baimaii *seems to be the main species in Myanmar, occurring throughout the country, then giving way to *An. dirus s.s*. eastward from western Thailand. *An. dirus s.s*. is the only species recorded so far in Vietnam, Lao, Cambodia [27-29] and Hainan Island [30]. *An. crascens *is confined to southern Thailand and Malaysia[31], and *An. takasagoensis *to Taiwan [32]. *An. scanloni *and *An. nemophilous *seem to have more patchy distributions. *An. scanloni *is restricted to western and southern Thailand, whereas the distribution of *An. nemophilous *closely follows monsoon forests. The south of Thailand and the Thai – Myanmar border present various sites of high sympatry for several species of the complex [3].

Some clarifications are still called for. Variations of ITS2 rDNA usually occur only between sibling species, thus intraspecific variations between populations of *An. baimaii *from Thailand and site 302 in China, and *An. scanloni *at sites 175 and 195 suggest the possible existence of two new species [15], with a less diverse population at site 195 indicating a period of isolation[33].

There is need for some investigations at the Thai-Cambodian border, the border between Lao and China and some areas in Myanmar. Indeed, although only *An. dirus s.s *seems to occur in Cambodia, two specimens of *An. baimaii *were earlier recorded near to the border in Thailand at site 241 [34] and specimens at the border have been observed to feed on monkeys in the canopy as would *An. nemophilous *in nearby Thailand [35]. Only *An. dirus s.s*. has been recorded in Lao although *An. baimaii *occurs nearby in China. Unexpected records such as *An. dirus s.s*. at site 49 [36] and 148 [37], and *An. scanloni *at site 126 [37], should be taken with caution and investigated further. This is also the case for records of *An. baimaii *at site 241, which are based on the collection of only two specimens. Sallum *et al*. [4] reported the presence of *An. crascens *in Sumatra but gave no reference.

### Larval habitats

Primary and secondary larval habitats of *An. dirus s.l*. have some constant characteristics: temporary, standing or slowly moving water under shade. While primary sites occur year round and tend to be associated with the stream system in deep forest, they vary in nature according to the season with drying pools in stream beds, pools connected with streams or meanders of slow moving streams in the dry season [38-40], complemented in the rainy season by pools fed by underground seepage along streams, springs, rock pools in the beds of ravines, and in deep holes or pits which form ahead of gullies [32,39,41]. Such primary sites are often confined to the deep forest resulting in transmission of malaria mainly to forest workers. Some of them however occur in peri-domestic area: *An. dirus s.s*. occurs in gem pits [42] and in water pits fed by seepage along streams [43]. If wells are generally negative[39,44], *An. baimaii *occurs in wells close to houses in a particular area of Myanmar throughout the year [36,45-47] even if densities are reduced in the dry season.

**Figure 4:**
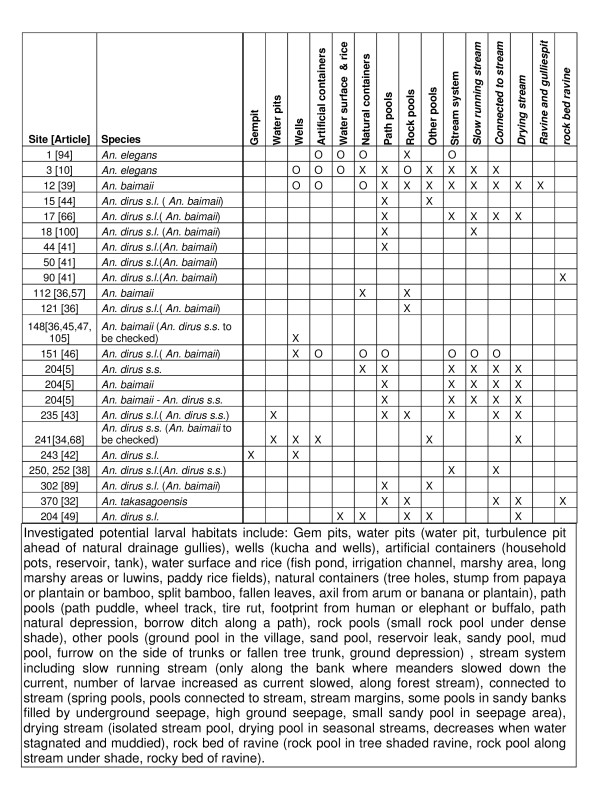
**Larval habitats for the *Anopheles dirus *complex**. Figure 4 shows for some sites and records, presence (X) or absence (O) in a particular larval habitat, as well as reviewed publication and the species identified, with probable species in brackets.

Secondary larval habitats occur in the rainy season and can be found closer to human settlement at the forest fringe. These are commonly small, shallow, temporary, shaded water-holding depressions [48]. Puddles in paths are commonly positive, whereas larger bodies of water such as ponds, large rivers, irrigation channels and marshy areas are generally negative. However, *An. takasagoensis *breeds in large permanent pools used by buffaloes for bathing if freshened by the rain [32]. *An. dirus s.l*. was also recorded in long marshy areas in the forest of Myanmar [41] and in two swamps and one rice paddy in Thailand[49]. Artificial containers [39] and natural containers are also generally negative but *An. dirus s.s*. is found reported in clay jars in Hainan[2], in a tin-hat[40], empty tins and in terracotta jars in Vietnam[50]. Positive natural containers include only bamboo stumps [5,36,40] and palm leaves [40]. Details on larval habitats and locations investigated are presented in Figure 4.

Immature stages of *An. dirus s.s*. and *An. baimaii *show adaptations to temporary habitats [39,43]. The eggs survive on the moist floor of a drained pool up to one month after the rain stops, but not throughout the dry season, unless sporadic rains prevent the soil from drying or unless the eggs are on the wet soils of forest. Not only do the eggs survive between rainfalls but they can also partially mature on a moist surfaces thus reducing development times, with first stage larvae present in pools containing no water prior to rain [39,43]. Larval stages can crawl to other pools in case of drying but they can also survive the desiccation of a draining pool and ant predation by getting covered in a coat of mud and becoming buried and motionless just before complete drainage. On re-flooding, only *An. dirus s.l*. reappears at the surface thus eliminating other competitors. *An. dirus s.l*. immature stages can feed on the larvae of other species and less frequently on its own larvae, in particular under crowded conditions [39]. Increased larval density results in higher larval mortality[36] and the production of smaller adults even with the same amount of food per larva, suggesting an influence on feeding efficiency rather than a shortage of food per se [51].

For a sibling species, larval habitats seem quite diverse according to location but Baimai [3] recorded a preference for vegetated limestone rock pools for *An. scanloni *and rocky-stony micro-environments for *An. dirus s.s*., *An. crascens *and *An. baimaii*. Sympatric populations of *An. dirus s.s*. and *An. baimaii *share identical larval habitat and even the same pools at site 204 [5]. *An. baimaii *was, however, more often found in pools that dried out in dry spells during the rainy season. This might reflect a seasonal variation in larval habitats or species, with *An. dirus s.s*. more abundant at the start and *An. baimaii *during the middle of the rainy season [3].

### Adult particularities and behaviour

*An. baimaii *and *An. dirus s.s*. are extremely anthropophilic [1,52-55], even in the presence of numerous cattle [56], but less anthropophilic behaviour has been sporadically recorded for these two species [27,57]. In particular, higher densities were recorded on cattle in some years [46] or every year for *An. baimaii *[58]. Some studies show that *An. nemophilous *feeds primarily on monkey, and *An. dirus s.s*. and *An. scanloni *feed more often on humans than *An. crascens*. These experiments were however based on few observations[3]. Monkeys are natural and winter hosts [2,32,59] but preference for monkey was not evident from human- and monkey-bait collections in the forest. More specimens were caught on human bait on the ground. As many were caught on monkey bait in the canopy as on human bait on the ground [35]. Anecdotal records report individual mosquitoes feeding on birds [56], dogs and pigs [41], as well as mixed feeding on bovines and humans suggesting that mosquitoes do not always come back to the same host[57,60,61].

Females are primarily exophagic but enter open shelters to feed [62]. Variations between regions are linked to housing facilities with the highest endophagy in largely open houses built directly on the ground [53]. Most open houses in the jungle show no significant biting differences between indoors and outdoors [1,56,57,63,64]. In some cases, indoor biting is even higher [38,43,65].

Resting places are difficult to find and occur mainly outdoors [58,59,66] in holes, wells[36,58,63]and vegetation such as bushes, tree holes [41,52], thick grass, under the surfaces of leaves [36,40], branches near the ground [40] and under the foots of trees[2]. In India, an intensive search collected none on the ground but 20 from the moist, dark crevices of large tree trunks, and 2 from creepers in the forest, the nearest at 150 m from a village[66]. Day-resting can be separated from night-resting[67]. Females rest early in the evening in outdoor vegetation, fences and wood stacked around houses [52,56] but after feeding, they fly almost immediately back to the jungle[1], with none found even at dawn around dwellings [43,52]. Eyles [35] caught more *An. dirus s.l*. in the canopy than on the ground and when Wilkinson [43] released hundreds of females in predawn darkness, they flew immediately upwards into trees suggesting that they might rest in the canopy of trees.

**Figure 5:**
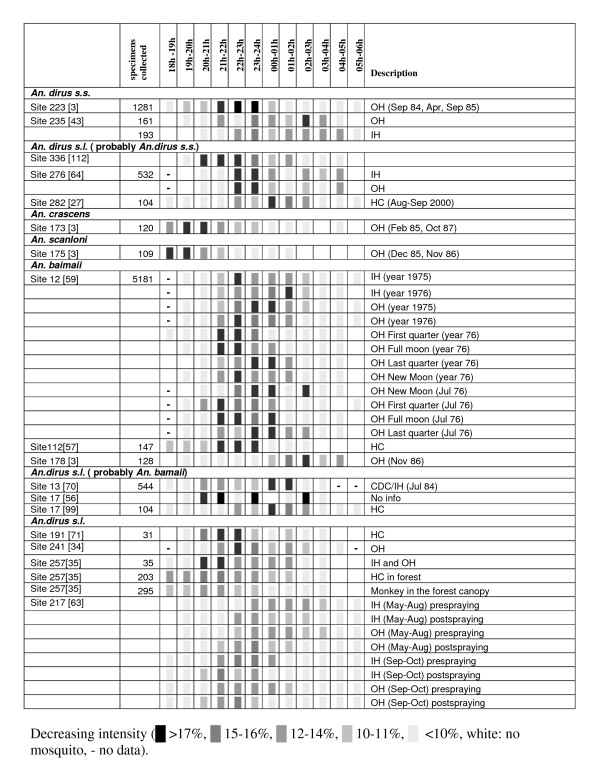
**Biting behaviour of species of the *Anopheles dirus *complex**. Figure 5 lists the hourly biting pattern of sibling species by collection sites (Site + number) and reviewed publication [reference]. The number of specimens collected (when available) gives an indication of the level of confidence. The data presented in the source article were converted to hourly percentages to allow comparison of data from different sources. A shaded symbol provides an easy reading of peak period and intensity of biting with decreasing density. Information on the types of collections, i.e. Outdoor Human collection (OH), Indoor Human collection (IH), Human collection (HC) and CDC light trap (CDC), is presented in the description section.

Biting can happen in daylight in the jungle[38,59] but occurs mostly from dusk to dawn. From the feeding patterns reported in the literature and presented in Figure 5, it is not obvious whether or not *An. dirus s.l*. is a late night feeder or an early night feeder. Scanlon [62] emphasised that late night feeding is most common in Thailand but that occasionally local populations exhibit a striking early pattern. The peak feeding activity has also been recorded either late [52] or early [68] in the same location for different years. Species-specific patterns of outdoor biting were suggested for allopatric populations with a very early peak for *An. scanloni*, and *An. crascens*, around 22h for *An. dirus s.s*. and 02h for *An. baimaii *[3]. *An. dirus s.s*. and *An. baimaii *are, however, both often recorded as late night feeders [1,2,27,35,48,69,70], but peak activity was also recorded well before midnight for *An. takasagoensis *and *An. baimaii *[32,41,46,57,71]. The occurrence of a particular sibling species can thus not explain all the variation. Other influencing factors seem to include the moon phase, the season and the presence of DDT, which stimulates early biting [59,63]. A two-year survey at site 12 recorded wide a repeated variation of the feeding pattern in accordance with the moon phase [59]. Variations have also been recorded between years and seasons [34,36,46,59] with earlier peak activity in the dry season[27] or earlier peak activity in October than in June [63]. Late biting in post-monsoon in some regions might also be due to the late onset of dawn [60]. Compilation of data across years, seasons, moon phases or between indoor and outdoor biting can thus result in a wide variety of patterns that are difficult to interpret [64]. The Relative Risk (RR) of being bitten in the hour before 22h compared with an hour after 22h probably provides a better indicator of exposure. A large proportion of bites before 22h coincide with activities of people before bedtime when the bed-net protection is nil [53]. Indoor biting peaks occur often later than outdoor biting [59] but in the presence of very open houses no difference is observed [53]. Outdoor biting has been recorded as starting as early as 16h for *An. scanloni *[3], and 19h for *An. baimaii, An. dirus s.s*. and *An. takasagoensis *[1,32,38,43,46,72].

### Environmental drivers

The occurrence of different sibling species can explain part of the heterogeneity in behaviour. However, differences between individuals of the same species underlines the major role of environmental factors in determining the occurrence, distribution, seasonality, behaviour and vectorial status for *An. dirus s.l*. The influence of moon phase and housing facilities on biting behaviour has already been discussed. Other keys factors of importance are temperature, rainfall, topography, season, soil type, shade, water quality and land-cover. These factors interact with components of the mosquito life cycle on one hand and with different stages of the parasite cycle on the other.

The occurrence of *An. dirus s.l*. is mainly linked to rainfall, temperature and relative humidity [46]. Attacks started after the rain exceeded 50 mm at site 12 for *An. baimaii *[39]. Lower or different thresholds seemed to apply according to the soil type and topography with, for example, *An .dirus s.l*. in Hainan [73]. Heavy rains however, flush away larval habitats, impede mosquitoes from flying [3,46,74] and the flooded grounds can be unsuitable for weeks[43]. The pattern of rainfall might, thus, be more important than the amount of rainfall, and light rains, not too frequent, seem to be most favourable for larval development [32]. Kitthawee [51] observed that adult body size was positively correlated with rainfall 1–2 weeks before. She suggests that renewed rainfall may bring more particulate food onto the surface of the water where larvae feed. Conversely, excessive rain could dilute nutrients and lead to nutritionally stressed, smaller adults. The importance of the rainfall pattern can explain yearly fluctuations in populations recorded during different studies in the same region [28,29,64]. The month of peak densities is not constant but usually takes place during the rainy season, with up to 80 bites per man/night [75]. Variations of relative abundance of sibling species in sympatric sites show more *An. crascens *than *An. baimaii *at the beginning and less *An. crascens *than *An. scanloni *at the end of the wet season at site 175. At site 204, more *An. dirus s.s*. were found at the start and more *An. baimaii *towards the middle of the wet season [3]. Populations of *An. takasagoensis *also present high fluctuations in abundance. The species usually is fairly uncommon but is occasionally very abundant [32].

Temperature is rarely a limiting factor but it influences the longevity of the mosquito, the length of the sporogonic cycle and mosquito activity, thus influencing seasonally the vector status.*An. dirus s.l*. seems inactive when temperature falls below 15°C [27]. However, in the deep forest of northern Thailand, during the cool, dry season associated with important temperature fluctuations and minima around 10°C, *An. dirus s.l*. can still survive and even transmit malaria. [63]. The duration of the immature stages is reduced at higher temperatures but females are then smaller [36]. If size does not influence the number of oocysts [76], larger mosquitoes live longer and have thus a greater vectorial capacity [77]. Mean daytime temperature averages 25.4°C with up to 5°C variation in positive ground pools [43]. However, in colonised wells temperature remains almost constant (26.2°C), despite outside temperature oscillations from 19.5 to 33.1°C. The clay contained in the lateritic wall lining the wells might have a sustained cooling effect[47].

Topography, salinity, pH and shade also influence the availability and occurrence of larval habitat. Topography is a major element with sites found commonly in foothills where rain water can accumulate, next to streams or in the beds of ravines. Rosenberg observed that the appearance of waves of *An. dirus s.l*. after the rain is not systematic at site 12 and requires three concomitant elements: rapidly draining pools, intermittent, heavy rains and embryonated eggs [39]. If suitable pools are temporary, they should hold water for at least five to eight days [39,66]. Suitability of a site might, thus, be a combination of the clay content of the soil with the appropriate amount and frequency of rain. In the sandy soils of site 12, suitable larval habitats occur only on the compacted soil of the path. The infiltration is too high elsewhere. Where *An. baimaii *breeds year-long in wells, the soil outside is not appropriate for larval habitat because it dries out very rapidly by percolation and evaporation. Even puddles in path are negative [46] and most wells dry up in summer [45]. Kitthawee [42] noticed that site such as 148 and 243 which presents the particular behaviour of year-long larval development in gem pits or wells also present a particular environmental setting with very high rainfall and infiltration rates.

Salinity, pH, shade and temperature have been extensively analysed in the context of *An. bamaii *in wells in Myanmar. Nitrate, iron, dissolved oxygen, sulphate, chlorine, ammonia and water hardness seem to have no influence, but larval density is negatively correlated to pH in ground pools and salinity in wells, with a threshold at 200 ppm NaCl [36,45,47,49]. Larval density becomes very low when the distance between the well-water surface and the ground surface is less than 2.5 m, probably reflecting an effect of shade and temperature [36,47]. Larvae gradually disappear if the shade is removed. Except for a few records [5,69], most larval habitats are under the shade of trees which probably reduces the drying speed of pools, influences temperature and provides food through organic matter and leaves falling into the water. Positive wells have shade and shrub on inner walls, debris on the water surface and often abundant decaying leaves at the bottom, even if the water is clean [36]. Numerous larvae are even observed in pools often fouled by buffaloes [2], but *An. dirus s.l*. will not be encountered if the water is not freshened frequently by rains [48].

### Association with forest

The most important environmental parameter is definitely land-cover. Throughout its distribution *An. dirus s.l*. is associated with forested foothills, forests or forest fringes. If availability of larval habitats and the presence of natural hosts, such as monkeys [35] are explanatory factors for this association, adults may also require the highest humidity and lower temperature of the jungle biotope for optimal survival [2]. Favourable environmental conditions of dense vegetation, humid soil, high relative humidity and shade, coupled with the presence of permanent suitable larval habitats or primary sites, appear to persist deep inside the forest during the dry season. Although rainfall in a tropical rain forest is unpredictable, the forest floor is humid. Even if the larval habitats loose their free water, the high humidity probably keeps the eggs viable for fairly long periods until the next rain comes[2]. The tree cover provides food for larvae, with leaves and other debris falling into larval habitats and assures stable micro-climatic conditions, even in the dry season. As the rainy season begins, conditions also become favourable at the margins of forest and *An. dirus s.l*. seems to spill over from the forest into secondary larval habitats.

Much larger densities of *An. dirus s.s*. are present in deep forest settlements or villages than in villages located at the edge of forest or in fragmented forests [78]. Variations in forest are difficult to interpret when mosquito densities are low near a village, very high at 1.5 km and moderate at 5 km[1,35,78,79], Infected mosquitoes can be present at oviposition sites 1.5 to 3 km away from the village [59]. Terracotta jars provided for oviposition in the forest attracted the highest number of mosquitoes at 150 m from the village but hardly any 300 m away [29]. Specimens were captured up to 2 km away from a source during a mark-release-recapture study [80]. These complex and contradicting results show that all the parameters influencing the occurrence and density of the *An. dirus s.l*. populations in the forest are not totally understood but the distance to the potential host and suitability of oviposition site certainly play a major role. The association with the forest is high in any case and almost systematically results in high vector contact and malaria transmission.

Species such as *An. nemophilous *seem to be dependant on monsoon forest[3]. In Cholbury, Thailand, large populations of *An. dirus s.l*. were encountered by Scanlon [52] in 1964, but the site has been further deforested and the population has been considerably reduced. However, adaptation to other land-cover has been recorded, particularly to teak and rubber plantations and orchards. The mosquitoes can adapt to the edge of man-made clearings [39,48,81] and have once been recorded in rice fields [49]. However, it is not known,, if large forested areas are necessary for the survival of these vector populations or if fragmented forest or plantations might be sufficient. The particular behaviour of year-long oviposition in wells might be a consequence of an adaptation to a new type of larval habitat and resting places that provide a cooler and more constant temperature, corresponding to the forest biotope [47].

### Vector status and control

The role played by *An. dirus s.l*. in the transmission of malaria has only been assessed during the last 50 years. It is now considered as the most important vector in Southeast Asia. Several factors contribute to making species of the *An. dirus s.l*. complex an exceptionally efficient vectors: they are so long lived and highly anthropophilic that only small populations are necessary to maintain high malaria endemicity [81]. Their exophilic behaviour, early biting habits and insecticide avoidance undermine the efficiency of the most common vector control measures e.g. insecticide residual spraying and insecticide impregnated nets. High human/vector contact in the typical forest biotope inhabited by species of this complex can explain the extended occurrence of what has been called "forest malaria".

The forest activities of humans play a major role in the malaria epidemiology of Southeast Asia [82]. The colonization of new land for agriculture, logging, mining and other activities, as well as resettlement of populations in the forest, expose people to high transmission risks in the most favoured biotope of *An. dirus s.l *Overnight stays in the open for hunting and collecting fruits in forest increase the human/vector contact, and open temporary shelters and forest huts facilitate early indoor biting when people are not yet protected by bed-nets[40]. The invasion of the jungle by human settlers most probably increases the densities of these mosquitoes by providing hosts and the small transitory pools that are preferred for oviposition [39]. During the dry season, people are mainly getting malaria in the deep forest [63] where infected vectors [43] are commonly found all year round near permanent streams [39]. Malaria attacks occur in villages only during the rainy season, when *An. dirus s.l*. moves back to the valley and forest fringe and where secondary larval habitats become available [59]. People move between villages and semi-permanent huts in forest and migrations between infected areas in the forest and non-infected areas trigger the start of transmission in the forest fringe and its surroundings once conditions there become favourable.

The vector density peak occurs generally one month before the malaria incidence peak [40]. However, large populations of mosquitoes are, not required for maintaining a high level of transmission [1,29,61,71]. Hence, small populations of mosquitoes might not be detected in short-term surveys as abundance and presence vary greatly between years, seasons and even from one week to another. In some regions, females feed late at night and may be missed unless night-long collections are made [62]. The sporozoite rates of *An. dirus s.l*. vary with season and location, with the highest rates recorded in October (7.8%) at site 17 [75] and rates up to 14% in forested site 22 [41]. Rosenberg [81] found high variation between villages 800 m away from each other with a sporozoite rate three to four times greater in the site of lower abundance. Sporozoites of *P.vivax *and *P.falciparum *have been commonly detected in *An. baimaii *and *An. dirus s.s *Baimai [83] reported sporozoites in *An. scanloni *and *An. crascens *with slight differences between species in relation to the parasite. *An*. *dirus s.s*. developed *Plasmodium vivax *and *P*. *falciparum *oocysts more readily than *An. crascens *and *An. scanloni*. *An. elegans*, *An. nemophilous*, and *An. takasagoensis *probably only transmit simian malaria [83].

Foci of chloroquine resistance have been commonly associated with *An. dirus s.l*. [1,2,70,84]. Wilkinson [85] carried out an experiment in a highly endemic area for chloroquine resistant strains of *P*. *falciparum *and showed that 66% of *An. dirus s.l*. and 44% of *An. minimus *became infected when fed on the same infected patients. When comparing the infected mosquitoes, the number of oocysts was also higher in *An. dirus s.l *Trung [29] recorded *P.falciparum*, *P.vivax*-210 and *P.vivax*-247 circumsporozoite protein (CSP) in a single *An. dirus s.l*. mosquito. The great longevity of *An. dirus s.l*., its high susceptibility to *Plasmodium *infections and a tendency to develop high numbers of oocysts increases the risk of recombining parasite strains in the mosquito gut and as consequence the risk for a fast spread of multi-drug resistance.

Alternative methods to human landing collection should be used where multi-drug resistance is present. CDC light traps have been used successfully on other species in Africa [86,87] and on *An. dirus s.l*. indoors in India [60] but other studies show less positive results when comparing various trapping method to human landing collections [88,89]. Alternative methods to human landing collection such as CDC light trap should be further evaluated as possible tool for monitoring vector control programmes.

*An. dirus s.l*. is susceptible to DDT [36,41,43,50] but due to exophilic behaviour, females avoid treated walls [43,52,63,90] or even avoid the sprayed huts by biting more outdoors after residual spraying [59,91] and high sporozoite rates may persist after application of DDT [2]. Insecticide impregnated bed-nets were proved to be effective [84] if kept in good condition [92], however, early biting habits in some areas exposes people to bites before bed time. Insecticide-treated hammocks and personal protection might thus be more effective. Alternative methods such as vegetation clearing are difficult to apply to such diffuse temporary larval habitats[2]. Treating the vegetation surrounding houses to target resting females would probably fail due to rapid loss of insecticide to rain and rapid vegetation growth [62].

## Conclusion

*An. dirus s.s and A. bamaii *of the *An.dirus *comple*x *are certainly the most efficient malaria vectors in Asia. Human activities in the jungle create high human/vector contact exposing people with poor shelter conditions in forested habitat, leading to perennial transmission. The efficiency of these species, as malaria vectors is largely explained by biological particularities. These species are highly susceptible to malaria parasites, there are highly anthropophilic, and have an excellent survival rate required for the sporogonic cycle. These vectors are difficult to control regarding the dispersion of temporary larval habitats in the forest, and their exophilic behaviour by which the mosquitoes entering the house will avoid any contact with indoor insecticide treated surfaces. Their relative early biting behaviour, preferably outdoors, may hamper the efficacy of insecticide treated nets (ITN). However, as these vectors are very sensitive to pyrethroids and almost exclusively anthropophilic, scaling up of ITNs will probably affect locally the *An.dirus s.l*. populations.

The distributions of species of the *An. dirus *complex have been thoroughly analysed in Thailand, Malaysia and recently in other areas of Southeast Asia following extended surveys from the Malvecasia project, but the distributions of members of the complex in the western region rely on few records and should be further investigated. Historical records and references from the literature have been used to delimitate the maximum extent of the complex distribution, but they do not reflect the current situation as major changes in land-cover have occurred in the region.

*An. dirus s.l*. is strongly associated with deep forest larval habitats and probably also requires deep forest for adult survival. It can survive year round wherever evergreen forest occurs. Drastic deforestation in recent decades has considerably reduced suitable habitats but adaptation of the species to man-made habitats such as orchards and plantations greatly increases the human/vector contact and suggests high plasticity in habitat requirements. However, such adaptations might only occur in areas where some of the environmental conditions, particularly micro-climatic conditions (e.g. wells, orchards) are still suitable.

High heterogeneity in behaviour has been recorded for mosquitoes of the *An. dirus *complex. The assumption that the recent discovery of seven cryptic species would explain most of this heterogeneity is challenged by the high behavioural differences recorded for different populations of the same species. *An. baimaii *specimens are developing in wells or forest habitats. Biting peaks vary from early to late within individual species and seasonality seems more linked to environmental factors than to species distribution. This could be a consequence of incomplete characterization of the group due to imperfect identification methods. Indeed, new molecular tools suggest the existence of two more species within the complex. Population history and phylogenetic relationships between the species are not straightforward and are sometimes even contradictory [33,93]. Behavioural differences between sibling species can only be analysed when they occur in sympatry, and very few sympatric populations have been studied. Allopatric species such as *An. dirus s.s*. and *An. crascens *are impossible to compare.

Environmental factors play a major role in intraspecific heterogeneity by interacting with the immature and adult stages. The most important factors are rainfall, which provides larval habitats and keeps growth conditions optimal by refreshing the sites and providing food, and the land-cover with conditions being are optimal year-long in the forest and seasonally in forest fringes. Temperature, topography, soil type, salinity and drainage also have an influence.

Environmental proxies might be relevant factors in a preliminary approach to establish approximate limits to the possible extension in the flexible distribution range of *An. dirus s.l*. However, variation in transmission dynamic occurs at very small spatial and temporal scales and can only be understood by studying micro-environmental parameters in details and in relation to human factors such as housing, settlement location in relation to the forest, occupations and migrations. This highlights the importance of micro-environmental variations on mosquito populations in a region that is currently undergoing major land-cover changes. Forest fragmentation and changes in land occupation influence habitat suitability for members of the complex. Although forest seems to be essential for mosquito survival, adaptations to orchards and wells have been recorded. Understanding the relationship between landscape components and mosquito population is thus a priority in foreseeing the impact of the land-cover changes on malaria occurrence and in shaping control strategies for the future.

## Authors' contributions

VO analysed the more than a hundred publications, designed the database of collection sites and the maps, and drafted the manuscript. PD contributed to the interpretation, presentation of the results and critically reviewed the manuscript. MC contributed his expertise in malaria vector control in Southeast Asia as well as the MALVECASIA database of more than a hundred newly investigated sites in Southeast Asia. He critically reviewed the manuscript. All authors read and approved the final manuscript.

## Supplementary Material

Additional file 1 Map Key. Correspondence between collection sites number and reviewed publications: sibling species and identification methods. Click here for file

Additional file 2 Collection sites description. The table lists 537 sites with geographical coordinates used for the display on the maps and true coordinates as well as details on mosquito collections and site description Click here for file

Additional file 3 Collection sites listed by reviewed publications. The table lists 130 reviewed publications with associated collection sites, species identified and identification method. Click here for file

Additional file 4 Abbreviations and descriptions used in the other additional files. lists information and description for each column and each table as well as abbreviations included in the file. Click here for file
