# Supplementary material for: The Anopheles dirus complex: spatial distribution and environmental drivers
Source: Malar J. 2007 Mar 6;6:26. doi: 10.1186/1475-2875-6-26 (PMC1838916; doi:10.1186/1475-2875-6-26)
Supplement: Additional file 1 — Map Key. Correspondence between collection sites number and reviewed publications: sibling species and identification methods. [file 1475-2875-6-26-S1.pdf]

Site[Article]	Species	Site[Article]	Species	Site[Article]	Species
1[94]	*An. dirus s.l.*[8]	80-81 [95]	No specimen	149 [58,95]	*An. dirus s.l.*[8]
2 [23]	*An. elegans*[1]	82 [95]	*An. dirus s.l.*[8]	150 [95]	No specimen
2 [22]	*An. dirus s.l.*[8]	83-89 [95]	No specimen	151 [46]	*An. dirus s.l.*[9]
3 [10]	*An. dirus s.l.*[8]	90 [41,95]	*An. dirus s.l.*[8]	152-155 [95]	*An. dirus s.l.*[8]
4-8 [10]	No specimen	91-93 [95]	No specimen	156 [95] [58]	*An. dirus s.l.*[8]
9 [25]	*An. dirus s.l.*[8]	94 [95]	*An. dirus s.l.*[8]	156 [36]	*An. baimai*[9]
10 [24]	*An. dirus s.l.*[9]	94-101 [95]	No specimen	157-159 [16]	*An. nemophilous*[7]
11 [54]	*An. dirus s.l.*[9]	101 [95]	*An. dirus s.l.*[8]	160 [4]	*An. crascens*[7]
12 [39,59,96]	*An. dirus s.l.*[8]	102-108 [95]	No specimen	161 [15]	*An. crascens*[4, 1]
12 [26]	*An. baimaii*[1]	109 [95]	*An. dirus s.l.*[8]	162 [62]	*An. dirus s.l.*[8]
12 [33,93]	*An. baimaii*[4]	110-111 [95]	No specimen	163-166 [62]	No specimen
12 [4]	*An. baimaii*[7]	112 [26]	*An. baimaii*[1]	167 [3]	*An. crascens*[1]
13 [70]	*An. dirus s.l.*[8]	112 [36,95]	*An. dirus s.l.*[8]	167 [97]	*An. crascens, An. nemophipous*[1]
14 [98]	*An. dirus s.l.*[9]	112 [57]	*An. baimai*[9]	167 [15]	*An. nemophilous*[4, 1]
15 [44]	*An. dirus s.l.*[8]	113 [95]	No specimen	167 [16]	*An. nemophilous*[7]
16 [84]	*An. dirus s.l.*[9]	113 [41,95]	*An. dirus s.l.*[9]	168 [M]	No specimen
17 [56,60,66,75,79,99]	*An. dirus s.l.*[9]	114-115 [95]	No specimen	169-170 [16]	*An. nemophilous*[7]
18 [65,69,100]	*An. dirus s.l.*[9]	115 [41,95]	*An. dirus s.l.*[9]	171 [4]	*An. crascens, An. scanloni*[7]
19-21 [95]	No specimen	116 [33,93]	*An. baimai*[4]	172 [62]	*An. dirus s.l.*[8]
22 [41,95]	*An. dirus s.l.*[8]	117 [95]	*An. dirus s.l.*[8]	173 [3]	*An. crascens, An. scanloni*[1]
23-24 [95]	No specimen	118 [95]	No specimen	174 [62]	No specimen
25-26 [101]	*An. dirus s.l.*[9]	119 [36,58,95]	*An. dirus s.l.*[8]	175 [3]	*An. crascens, An. scanloni, An. baimaii*[1]
27 [95]	*An. dirus s.l.*[8]	119-120 [95]	No specimen	175 [4]	*An. baimaii*[7]
28 [95]	No specimen	121 [36,58,95]	*An. dirus s.l.*[8]	175 [3]	*An. crascens, An. scanloni*[1]
29 [41,95]	*An. dirus s.l.*[8]	122 [36,95]	*An. dirus s.l.*[8]	175 [16]	*An. nemophilous*[7]
30 [41,95]	*An. dirus s.l.*[8]	123 [36,95]	*An. dirus s.l.*[8]	175 [15]	*An. scanloni*[4, 1]
30 [95]	No specimen	124-126 [95]	No specimen	175 [33,93]	*An. scanloni*[4]
31 [95]	*An. dirus s.l.*[8]	127 [37]	*An. scanloni*[1]	175-176 [16]	*An. nemophilous*[7]
31-34 [95]	No specimen	127 [36,95]	*An. dirus s.l.*[8]	177 [3]	*An. scanloni*[1]
35 [95]	*An. dirus s.l.*[8]	127 [102]	*An. dirus s.l.*[1]	178 [3]	*An. crascens, An. baimaii*[1]
36-37 [95]	No specimen	128-129 [95]	No specimen	178 [4]	*An. baimaii*[7]
38 [41,95]	*An. dirus s.l.*[8]	130 [95]	*An. dirus s.l.*[8]	179 [4]	*An. crascens, An. baimaii*[7]
39-40 [95]	*An. dirus s.l.*[8]	131 [95]	No specimen	180 [16]	*An. nemophilous*[7]
41 [95]	No specimen	132 [95]	*An. dirus s.l.*[8]	180 [4]	*An. baimaii*[7]
41 [36,95]	*An. dirus s.l.*[8]	133-136 [95]	No specimen	181 [3]	*An. crascens, An. baimaii*[1]
42-44 [95]	No specimen	137 [36,95]	*An. dirus s.l.*[8]	182 [3]	*An. baimaii*[1]
44 [41,95]	*An. dirus s.l.*[8]	138-140 [95]	No specimen	183 [M]	No specimen
45-48 [95]	No specimen	141-144 [95]	*An. dirus s.l.*[8]	184-185 [16]	*An. nemophilous*[7]
49 [36]	*An. dirus s.s.*[9]	145 [33,93]	*An. baimai*[4]	186 [3]	*An. dirus s.s., An. baimaii*[1]
49-50 [95]	*An. dirus s.l.*[8]	146 [95]	No specimen	187 [4]	*An. crascens, An. baimaii*[7]
50 [41,58,95]	*An. dirus s.l.*[8]	147 [41,95]	*An. dirus s.l.*[8]	188-189 [71,103,104]	*An. dirus s.l.*[8]
51-62 [95]	No specimen	148 [45,58,95]	*An. dirus s.l.*[8]	190 [71,103,104]	No specimen
62 [41,95]	*An. dirus s.l.*[9]	148 [26]	*An. baimaii*[1]	191-192 [71,103,104]	*An. dirus s.l.*[8]
63 [41,95]	*An. dirus s.l.*[8]	148 [37]	*An. dirus s.s.*[1]	192 [3]	*An. dirus s.s., An. baimaii*[1]
64 [95]	*An. dirus s.l.*[8]	148 [36]	*An. baimai*[8]	193 [15]	*An. dirus s.s., An. baimaii*[4, 1]
65-78 [95]	No specimen	148 [102]	*An. dirus s.l.*[8]	193 [33,93]	*An. dirus s.s., An. baimaii*[4]
79 [95]	*An. dirus s.l.*[8]	148 [47,105]	*An. dirus s.l.*[8]	194 [4]	*An. scanloni*[7]

Table 1(continued)

Site[Article]	Species	Site[Article]	Species	Site[Article]	Species
195 [4]	*An. baimaii*[7]	229 [15]	*An. dirus s.s.*[4, 1]	283 [27]	No specimen
195 [15]	*An. scanloni, An. baimaii, An. nemophilous*[4, 1]	229 [33,93]	*An. dirus s.s.*[4]	284 [27]	*An. dirus s.l.*[8]
195 [3]	*An. dirus s.s., An. scanloni, An. baimaii*[1]	230 [91]	*An. dirus s.l.*[8]	284-285 [M]	*An. dirus s.l.*[8]
195 [15]	*An. scanloni, An. baimaii*[4, 1]	231 [3]	*An. dirus s.s.*[1]	285 [27]	*An. dirus s.l.*[8]
195 [33,93]	*An. scanloni, An. baimaii*[4]	231 [15]	*An. dirus s.s.*[4, 1]	286 [27,106]	No specimen
195 [15]	*An. scanloni, An. baimaii*[4, 1]	231[33,93]	*An. dirus s.s.*[4]	287 [107]	*An. dirus s.l.*[8]
195 [16]	*An. nemophilous*[7]	232 [108]	No specimen	288-289 [28]	No specimen
195 [4]	*An. scanloni*[7]	233 [3]	*An. dirus s.s.*[1]	290-291 [M]	No specimen
195 [M]	*An. dirus s.l.*[8]	234 [16]	*An. nemophilous*[7]	292 [29,53]	*An. dirus s.s.*[6]
196-197 [4]	*An. scanloni*[7]	235 [43]	*An. dirus s.s.*[7]	292 [28]	No specimen
198 [15]	*An. baimaii*[4, 1]	236 [109]	*An. dirus s.l.*[9]	292 [M]	*An. dirus s.l.*[8]
199 [4]	*An. baimaii*[7]	237 [M]	No specimen	293 [M]	No specimen
200 [3]	*An. dirus s.s.*[1]	238-239 [16]	*An. nemophilous*[7]	294 [28]	*An. dirus s.l.*[8]
201 [3]	*An. dirus s.s., An. baimaii*[1]	240 [3]	*An. dirus s.s.*[1]	295-296 [M]	*An. dirus s.l.*[8]
202 [3]	*An. dirus s.s., An. baimaii*[1]	240 [M]	*An. dirus s.l.*[8]	297 [28]	*An. dirus s.l.*[8]
203 [15]	*An. dirus s.s., An. baimaii*[4, 1]	241 [34]	*An. dirus s.s., An. baimaii*[2]	298-300 [M]	No specimen
203 [33,93]	*An. dirus s.s., An. baimaii*[4]	241 [68,81,110]	*An. dirus s.l.*[8]	301 [74]	*An. dirus s.l.*[9]
204 [4]	*An. baimaii*[7]	242 [M]	*An. dirus s.l.*[8]	302 [30]	*An. baimaii*[5]
204 [49]	*An. dirus s.l.*[8]	243 [42,51,77]	*An. dirus s.l.*[8]	302 [89]	*An. dirus s.l.*[9]
204 [3]	*An. dirus s.s.*[1]	244 [15]	*An. dirus s.s.*[4, 1]	303-318 [M]	No specimen
204 [26]	*An. dirus s.s., An. baimaii*[1]	244 [33,93]	*An. dirus s.s.*[4]	319 [29,53]	No specimen
204 [5]	*An. dirus s.s., An. baimaii*[2, 3]	245 [16]	*An. nemophilous*[7]	319-326 [M]	No specimen
205 [16]	*An. nemophilous*[7]	246-249 [38]	No specimen	327 [M]	*An. dirus s.s.*[4]
206 [26]	*An. dirus s.s., An. baimaii*[1]	250 [38]	*An. dirus s.l.*[8]	328 [M]	*An. dirus s.l.*[8]
206 [15]	*An. dirus s.s.*[4, 1]	251 [M]	*An. dirus s.l.*[8]	329-330 [M]	No specimen
207 [111]	*An. dirus s.l.*[8]	252 [38]	*An. dirus s.l.*[8]	331-335 [M]	*An. dirus s.s.*[4]
207-208 [4]	*An. baimaii*[7]	253 [M]	No specimen	336 [112]	*An. dirus s.l.*[8]
209 [113]	*An. dirus s.l.*[9]	254-256 [M]	*An. dirus s.s.*[4]	336 [29,53]	*An. dirus s.s.*[6]
209 [61]	*An. dirus s.l.*[9]	257 [35]	*An. dirus s.l.*[8]	337 [M]	*An. dirus s.s.*[4]
209 [114]	*An. dirus s.l.*[9]	258 [M]	*An. dirus s.l.*[8]	338 [M]	No specimen
210 [3]	*An. dirus s.s., An. baimaii*[1]	259-263 [M]	*An. dirus s.s.*[4]	339 [M]	*An. dirus s.l.*[8]
210 [4]	*An. baimaii*[7]	264 [55]	*An. dirus s.l.*[8]	340-341 [M]	*An. dirus s.s.*[4]
211-213 [115]	No specimen	265 [M]	*An. dirus s.l.*[8]	341 [M]	*An. dirus s.l.*[8]
213 [3]	*An. dirus s.s., An. baimaii*[1]	266 [M]	No specimen	342-343 [M]	No specimen
214 [115]	No specimen	267 [29,53]	*An. dirus s.s.*[6]	344-345 [M]	*An. dirus s.s.*[4]
215 [116]	*An. dirus s.l.*[9]	267 [M]	*An. dirus s.l.*[8]	346 [29,53]	*An. dirus s.s.*[6]
216 [115]	No specimen	268 [M]	*An. dirus s.s.*[4]	346 [M]	*An. dirus s.l.*[8]
217 [63]	*An. dirus s.l.*[9]	269-271 [117]	*An. dirus s.l.*[8]	347-361 [M]	No specimen
218 [M]	*An. dirus s.l.*[8]	272 [M]	*An. dirus s.l.*[8]	362 [29,53]	No specimen
219-221 [3]	*An. dirus s.s.*[1]	273 [28]	*An. dirus s.l.*[8]	362-363 [M]	No specimen
222 [15]	*An. dirus s.s.*[4, 1]	274 [M]	No specimen	364 [73]	*An. dirus s.l.*[9]
223 [3]	*An. dirus s.s., An. baimaii*[1]	275-276 [M]	*An. dirus s.l.*[8]	365-366 [74]	*An. dirus s.l.*[9]
224 [3]	*An. dirus s.s.*[1]	276 [64]	*An. dirus s.l.*[8]	367 [118]	*An. dirus s.l.*[9]
224 [33,93]	*An. dirus s.s.*[4]	277 [28]	*An. dirus s.l.*[8]	368 [30]	*An. dirus s.s.*[5]
224 [15]	*An. dirus s.s.*[4, 1]	278-279 [28]	No specimen	369 [119]	*An. dirus s.l.*[9]
225-227 [3]	*An. dirus s.s.*[1]	280 [28]	*An. dirus s.l.*[8]	370 [32]	*An. takasagoensis*[8]
228 [M]	*An. dirus s.l.*[8]	281 [M]	*An. dirus s.l.*[8]		
229 [3]	*An. dirus s.s.*[1]	282 [27]	*An. dirus s.l.*[8]		

The Map key links the map and the reviewed publications and allows the reader to identify the correspondence between each collection site (Site) and the reviewed publications [Reference] in which the site was reported, including the sites collected by the MALVECASIA project [M]. Sibling species status as well as identification method are presented for the reader to evaluate the degree of confidence associated with each method, i.e. 1: polytene chromosomal banding patterns[17] , 2: DNA probes [19],3: enzyme electromorph [18], 4: ASPCR [15], 5: rDNA ITS2, 6: random amplified polymorphic DNA (RAPD) [20], 6: Taxonomic, 7: Morphologic, 8: Unknown. More details are available in the other additional files associated with this article.
